# Adipocyte Triglyceride Turnover Is Independently Associated With Atherogenic Dyslipidemia

**DOI:** 10.1161/JAHA.112.003467

**Published:** 2012-12-19

**Authors:** Keith Frayn, Samuel Bernard, Kirsty Spalding, Peter Arner

**Affiliations:** 1 Oxford Centre for Diabetes, Endocrinology & Metabolism, Churchill Hospital, Oxford, United Kingdom (K.F.); 2 Institut Camille Jordan, CNRS UMR 5208, University of Lyon, Villeurbanne, France (S.B.); 3 Department of Cell and Molecular Biology, Karolinska Institutet, Stockholm, Sweden (K.S.); 4 Department of Medicine, Karolinska Institutet, Karolinska University Hospital Huddinge, Stockholm, Sweden (P.A.)

**Keywords:** apolipoprotein, atherosclerosis, fatty acids, hyperlipoproteinemia, lipids

## Abstract

**Background:**

Inappropriate storage of fatty acids as triglycerides in adipocytes and their removal from adipocytes through lipolysis and subsequent oxidation may cause the atherogenic dyslipidemia phenotype of elevated apolipoprotein B levels and subsequent hypertriglyceridemia. We tested whether turnover of triglycerides in fat cells was related to dyslipidemia.

**Methods and Results:**

The age of triglycerides (reflecting removal) and triglyceride storage in adipocytes was determined under free living conditions by measuring incorporation of atmospheric ^14^C into these lipids within the adipocytes in 47 women and 26 men with a large interindividual variability in body mass index. Because limited ^14^C data were available, triglyceride age was also determined in 97 men and 233 women by using an algorithm based on adipocyte lipolysis, body fat content, waist‐to‐hip ratio, and insulin sensitivity. This cohort consisted of nonobese subjects since obesity per se is related to all components in the algorithm. Triglyceride turnover (age and storage) was compared with plasma levels of apolipoproteins and lipids. Plasma levels of apolipoprotein B and triglycerides were positively related to triglyceride age in adipocytes, when measured directly using radiocarbon analyses (*r*=0.45 to 0.47; *P*<0.0001). This effect was independent of subject age, waist circumference measures, and insulin sensitivity (partial *r*=0.29 to 0.45; *P* from 0.03 to <0.0001). Triglyceride storage showed no independent correlation (partial *r*=0.02 to 0.11; *P*=0.42 to 0.91). Algorithm‐based values for adipocyte removal of triglycerides were positively associated with plasma triglycerides and apolipoprotein B (*r*=0.44 to 0.45; *P*<0.0001) and (also positively) with the inflammation status of adipose tissue (*r*=0.39 to 0.47; *P*<0.05). These correlations were statistically independent of subject age and observed in men and women as well as in lean and overweight subjects when subgroups were examined separately.

**Conclusions:**

Decreased removal of adipocyte triglycerides (as indicated by a high triglyceride age in fat cells) is independently associated with circulating apolipoprotein B and triglycerides. This suggests a hitherto unknown role of triglyceride turnover in adipocytes for the development and/or maintenance of atherogenic dyslipidemia.

## Introduction

Dyslipidemia is a major factor behind atherosclerotic cardiovascular disease. Altered fatty acid metabolism in adipose tissue could lead to elevated circulating fatty acid levels and thereby cause atherogenic hyperlipidemia.^1–3^ Fatty acids stimulate apolipoprotein B (apoB) synthesis by the liver^4^ and thereby influence the circulating levels of triglycerides and small, dense low‐density lipoprotein particles.^5^ Elevation of circulating apoB (hyperapobetalipoproteinemia [or hyperapoB]) and triglycerides is a common atherogenic profile.^4–5^

**Figure 1.**
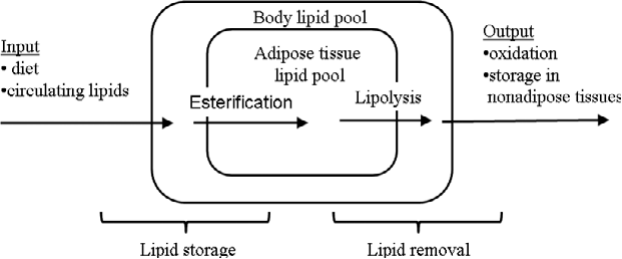
Adipose triglyceride turnover. A schematic diagram of parameters measured and derived. The primary measure is the average age of the adipose tissue triglyceride pool. The removal of triglycerides is governed largely by adipocyte lipolysis followed by oxidation and storage in nonadipose tissue; this pathway (“removal”) is the inverse of lipid age. The rate of lipid storage is calculated from body fat mass and lipid removal. It is largely dependent on the alimentary intake, circulating lipid levels and the capacity of adipocytes to synthesize triglycerides.

Storage and removal of triglycerides from fat cells (adipocytes) are key elements in the regulation of the energy balance. Whether these adipocyte properties are also important for the development of metabolic disorders has gained little attention, presumably due to previous lack of methods to study the turnover of adipocyte triglycerides under long‐term conditions. Imbalance in the triglyceride turnover in adipocytes may alter the shunting of fatty acids, the major structural components of triglycerides, through adipose tissue. A schematic view of lipid turnover is depicted in Figure 1. Fatty acids are taken up by adipocytes, mainly after hydrolysis of circulating triglycerides. Then, they are esterified to triglycerides within the adipocytes. The fatty acids are mobilized by intracellular hydrolysis (lipolysis) of adipocyte triglycerides and subsequently oxidized elsewhere. Changes in the storage and/or removal of the fatty acids influence the turnover of adipose triglycerides and may direct fluxes of fatty acids to nonadipose sites such as the liver.^1^ Although several factors may regulate the triglyceride turnover in adipocytes, some are of major importance (Figure 1). Lipid assimilation is controlled by alimentary intake, the levels of circulating lipids, and the capacity of fat cells to esterify fatty acids. The removal is dependent on the lipolysis capacity; storage of lipids in nonadipose tissues such as blood, muscle, and liver; and oxidation of the lipids.

We recently developed a method to determine triglyceride turnover in adipocytes in vivo during long‐term free‐living conditions.^6^ Atmospheric‐derived ^14^C incorporation into the triglycerides of adipose tissue is measured. Following atomic bomb testing in the mid‐1950s and early 1960s, there was a marked rise in atmospheric ^14^C, which exponentially declined after the bomb test treaty in 1963. The ^14^C from the atmosphere is taken up by plants. Because humans eat plants^7^ and some of the animals that live on them, the content of ^14^C in the atmosphere is directly mirrored in our bodies. By comparing ^14^C in triglycerides with ^14^C in the atmosphere over time, the age of these lipids in adipocytes can be determined. This reflects the removal of triglycerides from adipocytes by lipolysis and subsequent oxidation or (to a minor extent) storage of fatty acids outside of the adipose tissue.^6^ The triglyceride age data can be modeled when total body fat is known to also obtain an estimate of the rate of storage of triglycerides in adipocytes.

Using the described method, we found strong associations between adipocyte triglyceride turnover and metabolic conditions.^6^ In nonobese patients with familial combined hyperlipidemia (FCHL), both storage and removal of triglycerides were decreased in adipocytes. FCHL is the most common form of hereditary dyslipidemia and conveys a strong risk for developing coronary heart disease, as reviewed previously.^7–8^ The FCHL data support a pathophysiological model where a low triglyceride turnover in adipocytes causes an increase in the fatty acid flux to the liver so that hyperapoB and resulting dyslipidemia develop.^2,9^

Presently, we directly investigated how adipocyte triglyceride turnover parameters relate to the levels of different apolipoproteins and lipids in the circulation. In a smaller and heterogeneous sample of men and women with large interindividual variations in body mass index (BMI), insulin sensitivity, plasma lipids and apolipoprotein levels were investigated. In a larger and more homogeneous sample, an algorithm‐based measure of triglyceride turnover was used to assess its relation to the lipid profile.

## Methods

### Subjects

Clinical data are given in Table 1. In cohort 1 (26 men and 47 women), triglyceride turnover was investigated directly. The subjects were a subgroup of a cohort of 91 subjects previously investigated for adipocyte triglyceride turnover with the ^14^C method (see Supplementary Information 2 in Arner et al^6^ for details on the whole cohort). The subgroup was chosen since we had data on plasma lipids and apoliproproteins on the included subjects. Thirteen subjects had FCHL, which was diagnosed as we described previously.^10^ Two of these patients were receiving a fibrate or a statin. The remaining subjects with FCHL received no treatment at the time of investigation. All subjects with FCHL were nonobese (BMI <30 kg/m^2^). Among the remaining subjects, 4 had essential hypertension (1 concomitant with type 2 diabetes and 1 concomitant with unclassified and untreated dyslipidemia). Hypertension was treated with an angiotensin‐converting enzyme inhibitor and/or a calcium blocker. Four subjects had type 2 diabetes treated with diet and metformin. The remaining subjects were healthy and free of continuous medication. Among the subjects without FCHL, 37 were nonobese and 23 were obese. A second cohort of nonobese subjects (233 women and 97 men) was also investigated and triglyceride turnover was determined using an algorithm. All these subjects were nonobese because all the components in the algorithm were strongly associated with obesity as shown previously.^6,10^ This population‐based cohort was recruited by local advertising. Inclusion criteria were that they never had been obese and that they were healthy according to self‐report. Ninety‐nine of these subjects were overweight (BMI >25 to <30 kg/m^2^). The algorithm was based on data published previously.^6^ All subjects were weight stable for at least 6 months before examination according to self‐report and none had a history of marked weight reduction. Height, weight, and circumferences of waist and hip were measured and overnight fasting venous plasma levels of insulin, glucose, triglycerides, total and high‐density lipoprotein cholesterol, and apolipoprotein A1 (apoA1) and apoB were determined through the hospital's accredited routine clinical chemistry laboratory. Body fat content was measured with bioimpedance using BodyStat Quad Scand 400 (BodyStat Ltd, Isle of Man, British Isles). Insulin resistance was determined by calculating the homeostatic model assessment–insulin resistance (HOMA‐IR) index from insulin and glucose values as described previously.^11^ Finally, an abdominal subcutaneous fat biopsy sample was obtained as a needle biopsy specimen under local anesthesia^6^ for measures of lipolysis and triglyceride turnover. Lipolysis was measured in freshly isolated adipocytes as described previously.^6^ In brief, fat cells were isolated via collagenase treatment and incubated in vitro for 2 hours at 37°C in the absence or presence of 10^−10^ to 10^−6^ mmol/L isoproterenol (a synthetic catecholamine). At the end of incubation, medium glycerol (lipolysis index) was determined. There is no consensus how to express lipolysis data (per total weight of cells, per number of cells, or as a ratio of basal glycerol release). Glycerol release at maximum effective isoproterenol concentration divided by basal glycerol release was presently used because it was better fitted to adipocyte triglyceride age in the algorithm than was glycerol release per number or total weight of fat cells. In a subset of the subjects from cohort 2 (n=30), adipokine secretion from pieces of the adipose tissue was also measured exactly as described earlier^12^ and was related to the lipid weight of the sample.

**Table 1.** Clinical Characteristics

Measure	Cohort 1 (26 Men and 47 Women), Mean (Range)	Cohort 2 (97 Men and 233 Women), Mean (Range)
Age, y	38 (22 to 62)	39 (18 to 72)
BMI, kg/m^2^	29 (20 to 53)	24.0 (17.8 to 29.9)
Body fat,%	37 (16 to 74)	29 (8 to 51)
Waist‐to‐hip ratio	0.94 (0.71 to 1.21)	0.89 (0.71 to 1.08)
apoB, g/L	1.1 (0.5 to 2.1)	0.9 (0.3 to 2.0)
apoA1 g/L	1.4 (0.6 to 2.9)	1.4 (0.8 to 2.9)
P‐triglycerides, mmol/L	2.2 (0.4 to 20.0)	1.4 (0.3 to 20.0)
P‐total cholesterol, mmol/L	5.7 (2.6 to 9.8)	4.8 (2.6 to 12.8)
P‐HDL cholesterol, mmol/L	1.3 (0.5 to 2.9)	1.5 (0.6 to 2.9)
HOMA‐IR index	2.7 (0.7 to 25.6)	1.4 (0.2 to 20.3)
Lipolysis, isoproterenol/basal	—	9.9 (1.6 to 36.1)
Adipocyte triglyceride age, y	1.9 (0.1 to 4.0)	1.41 (0.02 to 3.84)
Adipocyte triglyceride uptake, kg/y	24 (5 to 129)	—

BMI indicates body mass index; Apo, apolipoprotein; P, fasting plasma; HDL, high‐density lipoprotein; HOMA‐IR, homeostatic model assessment–insulin resistance.

Ethical approval was granted by the ethics committee at Karolinska Institutet, Stockholm, Sweden. Informed consent was obtained from all subjects.

### Extraction of Lipids From Adipose Tissue Preparations

Samples were stored at −70°C. Some samples consisted of pieces of intact adipose tissue, others were adipocytes isolated from fat tissue as described earlier.^13^ Methodological experiments revealed that ^14^C data from fat pieces and isolated adipocytes gave identical results.^6^ The samples were homogenized and lipid extracted as described.^6^ The resulting lipid sample was processed for ^14^C measurements.

### Carbon Isotope Measurements

Carbon isotope ratio measurements using accelerator mass spectrometry was performed exactly as described.^6^ ^12^C, ^13^C, and ^14^C were determined. Data were presented as the ratio of the ^14^C to ^12^C carbon for a sample divided by that of a well‐defined standard sample, measured in the same year, and incorporating ^13^C fractionation correction as described.^14^ Data were thereafter subjected to calculation of lipid age exactly as described.^6^ The turnover of adipocyte lipids in vivo was estimated using measures of ^14^C in adipose lipids set in relation to the atmospheric radioactivity.^6^ Adipocyte storage of triglycerides (kg/y) and adipocyte triglyceride age (reflecting removal and subsequent oxidation) were estimated using mathematical models.^6^ Values for triglyceride age and storage for each subject examined were obtained from a study published previously.^6^

### Statistics

Results were analyzed by single, multiple, or polynomial regression analysis and by ANCOVA. Correlation coefficients and partial correlation coefficients were determined from the single and multiple regression analyses. Confounders in multiple regression analysis and ANCOVA were age, sex, BMI, waist circumference, and HOMA‐IR. When several dependent parameters were tested simultaneously in Table 2 the *P*‐values were corrected according to Bonferroni (*P*‐value × the number of dependent factors). Values for HOMA‐IR, plasma triglycerides, in vitro lipolysis secretion of tumor necrosis factor‐α, and triglyceride storage were log_10_ transformed to improve normalization of distribution.

**Table 2.** Correlation Between Adipocyte Triglyceride Turnover and Plasma Lipids or Lipoproteins in Cohort 1

Independent Factors	Dependent Factors									
	P‐Triglyceride		P‐Total Cholesterol		P‐HDL‐Cholesterol		P‐apoA1		P‐apoB	
	*r*	*P* Value	*r*	*P* Value	*r*	*P* Value	*r*	*P* Value	*r*	*P* Value
Adipocyte triglyceride age	0.45	0.0002	0.31	0.012	−0.28	0.023	−0.24	0.04	0.47	<0.001
Adipocyte triglyceride storage	−0.21	0.13	−0.39	0.003	0.01	0.93	−0.14	0.28	−0.33	0.008

P indicates fasting plasma; HDL, high‐density lipoprotein; Apo, apolipoprotein.

## Results

We first made a single regression analysis for absolute measures of adipocyte triglyceride turnover parameters versus apolipoproteins (apoB and apoA1) or plasma lipids (Table 2). Both apolipoproteins and all of the plasma lipids (triglycerides, total cholesterol, and high‐density lipoprotein cholesterol) correlated significantly with triglyceride storage and/or triglyceride age with nominal *P*‐values <0.05. Regarding the significant correlation in Table 2, the following ones were positive: adipocyte triglyceride age versus plasma triglycerides or apoA1. All the remaining significant correlations for triglyceride age and for adipocyte triglyceride storage were negative. However, we also made a correction of the *P*‐values for multiple testing (Bonferroni, *P*‐value × 5). By this correction, all the correlations for apoA1 and high‐density lipoprotein cholesterol became nonsignificant and these parameters were consequently excluded from further analysis.

**Figure 2.**
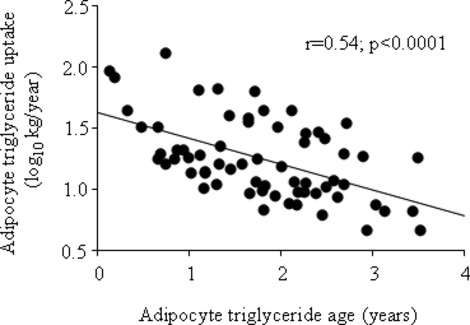
Relationship between triglyceride age and triglyceride uptake in adipocytes.

Adipocyte triglyceride storage and triglyceride age were significantly and inversely correlated (*r*=−0.54; *P*<0.001; Figure 2). This is expected because triglyceride storage values are derived from the triglyceride age values together with body fat mass.^6^ We therefore considered the various in vivo turnover parameters together in the next correlation analysis (Table 3). This analysis showed that the only remaining significant and independent correlations (positive ones) with turnover were adipocyte triglyceride age versus apoB and adipocyte triglyceride age versus plasma triglycerides (partial *r*=0.42 to 0.44; *P*=0.003 to 0.004). Therefore cholesterol was excluded from further analysis.

**Table 3.** Multiple Regression for Various Triglyceride Turnover Parameters Versus Circulating Factors in Cohort 1

Regressor	Triglyceride Uptake*		Triglyceride Age*	
	Partial *r*	*P* Value	Partial *r*	*P* Value
P‐apoB	−0.11	0.42	0.41	0.003
P‐triglycerides	0.02	0.91	0.44	0.004
P‐total cholesterol	−0.27	0.06	0.23	0.11

P indicates fasting plasma; Apo, apolipoprotein.

* Controlled for triglyceride age.

* Controlled for triglyceride uptake.

To study the influence of clinically important confounding factors, multivariate analyses were performed for adipocyte lipid age versus apoB or plasma triglycerides (Table 4). Triglyceride age was an independent and significant positive regressor for apoB and plasma triglycerides using any confounder as covariate variable (age, gender, BMI, waist circumference, or the insulin sensitivity index HOMA‐IR).

**Table 4.** Influence of Age, Gender, BMI, Waist Circumference, or HOMA‐IR on the Positive Relationship Between Adipocyte Triglyceride Age and Plasma Triglycerides or apoB in Cohort 1

Dependent Factors	Independent Cofactors*									
	Age		Gender		BMI		Waist Circumference		HOMA‐IR	
	Partial *r*	*P* Value	*F*	*P* Value	Partial *r*	*P* Value	Partial *r*	*P* Value	Partial *r*	*P* Value
Plasma triglycerides	0.43	0.0007	20.7	<0.0001	0.42	0.0009	0.39	0.003	0.29	0.032
Plasma apoB	0.42	0.0003	23.5	<0.0001	0.46	0.0001	0.45	0.0003	0.41	0.001

BMI indicates body mass index; HOMA‐IR, homeostatic model assessment–insulin resistance; Apo, apolipoprotein.

* Results with adipocyte triglyceride age as primary independent factor are shown. ANCOVA was used for age and multiple regression for remaining factors.

**Figure 3.**
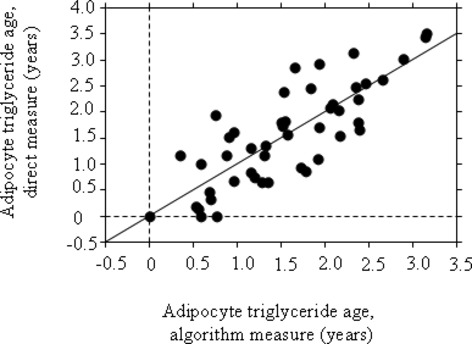
Indirect measure of adipose triglyceride age by an algorithm‐based equation is strongly correlated with directly measured lipid age, yielding a slope of 1 and zero intercept. The regression line is shown.

Cohort 1 was too small for investigating subgroups separately. Furthermore, the ^14^C dating method is resource demanding and time consuming. We therefore constructed an algorithm for triglyceride age (the most important single turnover parameter in this study) to be able to investigate a large group of nonobese subjects (cohort 2). In a previous study,^6^ we observed a strong relationship between triglyceride age on one hand and catecholamine‐induced lipolysis, body weight and body shape parameters, and insulin sensitivity (HOMA‐IR) on the other hand. We tested these parameters and several other measures in a multiple linear regression analysis to create a triglyceride age equation. We used all available data on 46 nonobese subjects in the previous study.^6^ The best‐fitting model (adjusted *r*^2^=0.63) is shown in Table 5 using percentage body fat, waist‐to‐hip ratio, HOMA‐IR, and isoprenaline‐stimulated lipolysis. Age or sex did not contribute significantly. Based on the coefficient data in Table 5 the following equation for triglyceride age (Y) was obtained: Y=−2.25−0.96×(10‐log isoprenaline/basal lipolysis)+0.056×(percentage body fat)+1.236×(10‐log HOMA‐IR)+3.182×(waist‐to‐hip ratio). This equation has a relationship between estimated and actually measured triglyceride age with slope=1.001 and intercept=0.007 (Figure 3).

**Table 5.** A Multiple Regression Model to Create an Equation From Which Adipocyte Triglyceride Age Can Be Estimated in Nonobese Subjects

Parameter	Coefficient	Standard Error	Standard Coefficient	*P*
% Body fat	0.056	0.013	0.399	0.0001
Waist‐to‐hip ratio	3.182	1.304	0.276	0.019
HOMA‐IR	1.236	0.39	0.324	0.003
Lipolysis	−0.96	0.338	−0.335	0.007
Intercept	−2.25	1.489	−2.25	0.138

Data are for the correlation between all regressions put together and adipose tissue triglyceride age measured by the ^14^C method in 46 nonobese subjects and obtained from a previous study.^6^ For homeostatic model assessment–insulin resistance (HOMA‐IR) and lipolysis (isoprenaline/basal), log_10_ transformed values were used.

Apo indicates apolipoprotein.

The algorithm was used to study triglyceride age in cohort 2. The age values correlated strongly (*P*<0.0001) and positively with plasma triglycerides in all subjects (*r*=0.47) and in men (*r*=0.53) or women (*r*=0.39). Also, apoB correlated strongly and positively (*P*≤0.0001) with adipocyte triglyceride age in all subjects (*r*=0.43), as well as in men (*r*=0.54) or women (*r*=0.41). The relationships were independent of subject age (partial *r*=0.35 to 0.46; *P*<0.0001) and found in either lean (n=209; *r*=0.35 to 0.40; *P*<0.0001) or overweight (n=121; *r*=0.48 to 51; *P*<0.0001) subjects.

In all nonobese subjects from cohort 1 where plasma triglycerides and apoB were determined, we compared the use of either the direct ^14^C method or the indirect algorithm method to measure adipocyte triglyceride age. Using either method, similar results were obtained with regards to the relationship with plasma triglycerides or ApoB (Table 6).

**Table 6.** Relationship Between Adipocyte Triglyceride Age Determined by Algorithm or Directly Measured Using Radiocarbon Dating and Plasma Levels of Triglycerides or ApoB*

Adipocyte Triglyceride Age	Plasma apoB (n=47)		Log Plasma Triglycerides (n=48)	
	*r*	*P* Value	*r*	*P* Value
^14^C method	0.50	0.0003	0.44	0.002
Algorithm	0.58	<0.0001	0.65	<0.0001

* Data are from nonobese subjects in cohort 1. Apo indicates apolipoprotein.

**Figure 4.**
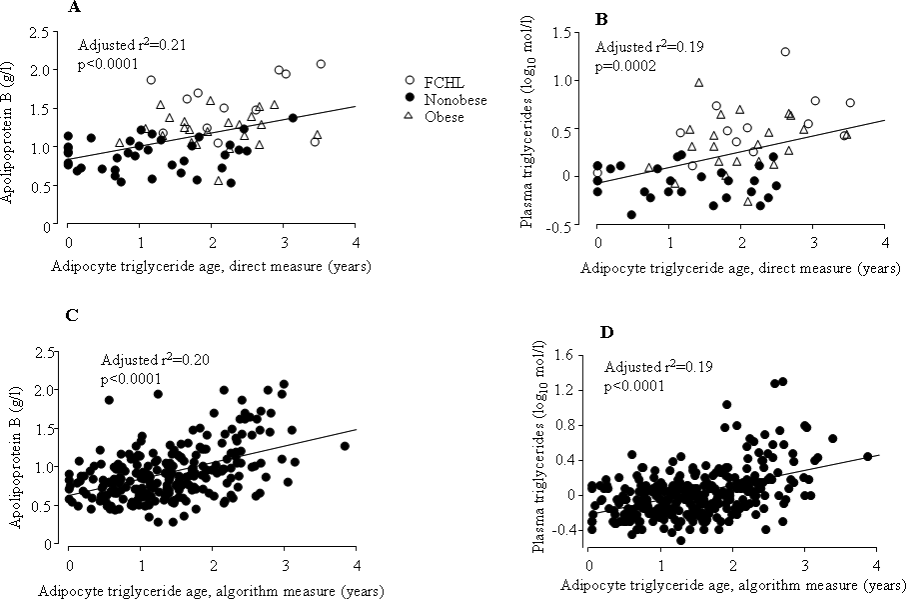
Relationship between adipocyte triglyceride age and ApoB (A, C) or (log) plasma triglycerides (B, D) when the age was determined directly (A, B) in one cohort (cohort 1) or by an algorithm (C, D) in another cohort (cohort 2). FCHL indicates familial combined hyperlipidemia.

The relationships between plasma apoB or triglycerides and adipocyte triglyceride age is shown graphically (Figure 4). Triglyceride age of adipocytes measured directly or indirectly could explain as much as 20% of the variation in plasma triglycerides or apoB (adjusted *r*^2^ in either cohort). Using the same type of calculation insulin sensitivity, BMI, waist–hip parameters, age, or body fat content when examined alone could explain only 1% to 13% of the variation in plasma triglycerides and 1% to 15% of the variation in apoB.

**Figure 5.**
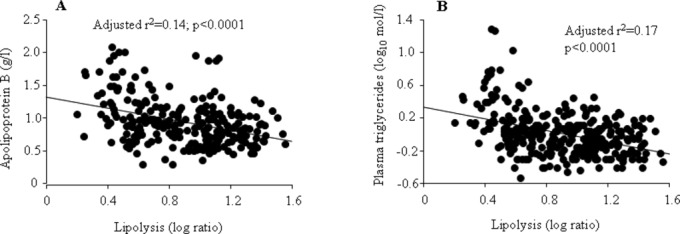
Relationship between adipocyte lipolysis (10‐log of isoprenaline stimulated/basal rate of glycerol release) and apoipoprotein B (A) or (10‐log) plasma triglycerides (B).

Because adipocyte lipolysis was a strong regressor for triglyceride age, we investigated its relationship with circulating lipid parameters. Lipolysis was strongly and inversely correlated with levels of both apoB and plasma triglycerides (Figure 5). These relationships were independent of BMI, percent body fat waist‐to‐hip ratio, HOMA‐IR, sex, and age in multiple regression analysis (partial *r* from 0.3 to 0.42; *P=*0.0002 to <0.0001).

**Figure 6.**
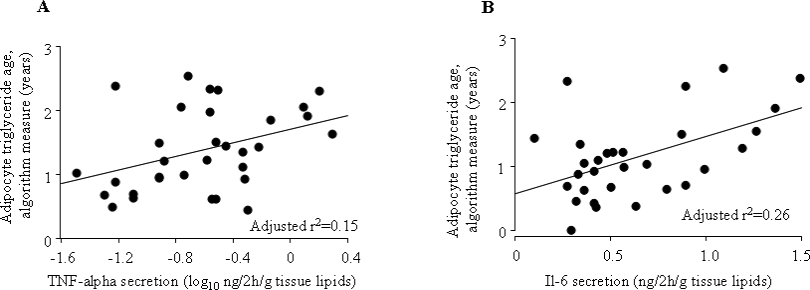
Relationship between adipose triglyceride age (determined by algorithm) and the secretion of tumor necrosis factor (TNF)‐α (A) or interleukin‐6 (IL‐6) (B) from adipose tissue.

The relationship between adipokine secretion from adipose tissue and triglyceride age was investigated in a subset of subjects in cohort 2 (Table 7). Neither leptin nor plasminogen activator inhibitor‐1 secretion in vitro correlated with adipocyte triglyceride age. However, the latter parameter showed a significant positive relationship with the secretion of tumor necrosis factor‐α and interleukin‐6. The linear relationships are depicted in Figure 6.

**Table 7.** Correlation Between Adipokine Secretion and Adipocyte Triglyceride Age Determined With Algorithm in Cohort 2

Adipokine	n	*r*	*P* Value
Leptin	43	0.04	0.81
Tumor necrosis factor‐α*	32	0.39	0.028
Interleukin‐6*	30	0.51	0.004
Plasminogen activator inhibitor‐1	44	0.11	0.50

* Due to lack of material, these adipokines could not be measured in all samples.

## Discussion

Although there is strong evidence for cholesterol as a risk factor for atherosclerotic cardiovascular disease, the role of triglycerides has been controversial. Recent evidence supports a role of both fasting and postprandial hypertriglyceridemia as independent and important cardiovascular risk factors.^15^ A recent meta‐analysis identified triglyceride‐mediated pathways as etiological factors behind coronary heart disease.^16^ HyperapoB is closely linked to disturbed fatty acid and triglyceride metabolism^17^ and is a strong risk factor for coronary heart disease.^18^

Earlier in vitro studies suggest that disturbed adipocyte triglyceride metabolism may be involved in atherogenic dyslipidemia, as reviewed previously.^2,19–20^ However, no immediate relationship has been demonstrated, although triglyceride synthesis and lipolysis in adipose tissue biopsies removed from patients with hyperapoB or FCHL were reduced compared with normolipidemic controls.^21^ Here we provide strong evidence that the turnover of triglycerides in adipocytes is involved in atherogenic dyslipidemia. When turnover was measured directly in a small patient cohort, the triglyceride removal rate (as measured by triglyceride age) was more important than the uptake rate for the relationship with plasma triglycerides and apoB. Furthermore, the relationship was independent of a number of confounding factors such as age, gender, BMI, waist circumference, and insulin sensitivity.

Findings with direct measures of triglyceride turnover were made in a cohort that was heterogeneous and too small for subgroup analysis. Therefore, we also measured triglyceride removal in a large homogeneous cohort of nonobese subjects by constructing an algorithm for triglyceride age based on major parameters influencing this turnover parameter. The algorithm measured triglyceride age and showed a good fit to the actual measure. In the large cohort, we also found a strong positive correlation between triglyceride age and plasma triglycerides or apoB lipoprotein, which was found in either sex and in lean or overweight subjects. Besides adipocyte lipolysis, the algorithm takes into consideration insulin sensitivity, body shape, and body fat, which influence the relationship between triglyceride age and the lipid profile. Adipocyte triglyceride age, when examined alone, could explain as much as 20% of the between‐individual variation in plasma triglycerides and apoB. This is higher than for common factors such as age, body shape, insulin sensitivity, and body fat measures. In cohort 2, each of these factors explains 1% to 15% of the variation.

The mechanisms by which triglyceride turnover in adipocytes influence circulating apoB and triglycerides are unknown but some speculation can be offered. We found that adipocyte uptake removal of triglycerides was strongly interrelated. Thus, a low removal is accompanied by low uptake. A low turnover of triglycerides in adipocytes may uncouple the adipose tissue from circulating fatty acids so that fatty acids are instead shunted to the liver, resulting in increased synthesis of both triglycerides and apoB. This has been suggested previously and attributed to a dysfunction of the acylation‐stimulating protein pathway.^9^ Although our data give no information on acylation stimulating protein, the present findings with algorithm based triglyceride removal are supportive of the uncoupling theory because the algorithm has lipolysis in the equation. Recently, the disturbed triglyceride metabolism in obesity has been attributed to a downregulation of fatty acid metabolic pathways in adipose tissue,^22^ which would accord with our data.

It is well known that obesity is a factor behind combined dyslipidemia. However, our findings are clearly independent of the body fat status. First, we only examined nonobese individuals in cohort 2. Second, results were the same when subjects in cohort 2 were subdivided into a lean (BMI <25 kg/m^2^) and an overweight group (BMI >25<30 kg/m^2^). Third, the relationship was independent of obesity in cohort 1.

Adipocyte lipolysis is a major factor in lipid mobilization and was a strong negative regressor for triglyceride age in this study as well as in our previous study.^6^ We presently observed that lipolysis strongly and inversely correlates with apoB and plasma triglycerides. This suggests that lipolysis in itself could be important for developing hyperapoB and elevated plasma triglycerides. It should be noted, however, that lipolysis is just one of many factors (presented in Figure 1) that determine adipocyte triglyceride age.

There are some caveats with our study limiting the pathophysiological conclusions. First, it is a cross‐sectional study (albeit the first report of its kind), which makes it difficult to say if triglyceride turnover is a primary or secondary factor. It is possible that disturbances in the turnover might cause atherogenic dyslipidemia. However, studies in humans and mice suggest that apoB can directly inhibit adipocyte lipolysis, which is a major factor in triglyceride removal.^23^ Thus, there may be cross‐talk between the liver and adipose tissue that could be of importance for adipocyte triglyceride turnover, such that liver production of apoB might regulate the lipid removal from adipose tissue. However, triglyceride turnover in adipocytes is a net function of uptake and removal of triglycerides. Other yet to be established factors determine the uptake. Second, we only investigated a single fat depot. Regional variations in lipid storage and removal from adipose tissue are known to exist as reviewed.^24^ Further prospective studies and investigations on different fat depots (subcutaneous, visceral) are needed to fully understand the importance of adipocyte triglyceride turnover for development and maintenance of atherogenic dyslipidemia. Such studies will necessarily take a long time. Because of the triglyceride half‐life in adipocytes, it is necessary to have a time gap of about 4 years between a first and second examination. Nevertheless, the present and previous data^6^ suggest a hitherto unrecognized pathophysiological role of adipocyte turnover of triglycerides in common and clinically important dyslipidemia disorders.

The endocrine function of adipose tissue, which is mediated by the production of so‐called adipokines, has emerged as a putatively important pathophysiological factor in many metabolic disorders.^25–26^ It is possible that inflammatory adipokines are involved in the regulation of adipocyte triglyceride turnover. We measured 4 adipokines in a subset of subjects. Whereas neither leptin nor plasminogen activator inhibitor 1 was related to triglyceride age, a positive correlation was observed for both inflammatory proteins investigated, namely interleukin‐6 and tumor necrosis factor‐α. A role of local inflammation in the normal control of lipid storage in human adipose tissue was recently suggested.^27^ It is possible that this involves the influence of adipose inflammatory proteins on adipocyte triglyceride turnover. Also, in this respect, there might be regional differences because the production and secretion of adipokines differ between subcutaneous and visceral adipose tissue.^28^

Several factors may influence circulating triglycerides, such as medication or lifestyle/dietary habits (not recorded here), and may have increased the variability in triglycerides measurements. However, it should be noted that increased variability would work against our findings and therefore strengthen their importance.

Although altered endocrine signals from adipose tissue may cause disease, it seems likely that lipid metabolism within the adipocytes is also involved, at least in dyslipidemia. We therefore propose adipocyte triglyceride turnover as a novel therapeutic target for hypertriglyceridemia and hyperapoB. However, both triglyceride uptake and removal should be considered together when targeting hypertriglyceridemia for the following reasons: Triglyceride removal is strongly linked to lipolysis in adipocytes.^6^ Nicotinic acid and its analogues lower plasma triglyceride at least in part by inhibiting adipocyte lipolysis^29–30^ but adrenoceptor blockers (also antilipolytic) may increase plasma triglycerides.^31^ Fatty acid oxidation is the ultimate pathway for their removal and it is of interest that the fibrate drugs, the most potent agents for lowering elevated triglyceride concentrations, act at least in rodents via upregulation of hepatic fatty acid oxidation pathways.^32^ In other words, the ways in which triglyceride turnover can regulate plasma lipids and apolipoproteins may be complex. Both inhibition and stimulation of one specific turnover pathway such as triglyceride removal from adipocytes may lead to the same phenotype (eg, hypertriglyceridemia) because removal and storage of triglycerides in adipocytes are strongly interrelated.

In summary, adipocyte removal of triglycerides is independently associated with an atherogenic hypertriglyceridemia/hyperapoB profile in 2 independent populations and explains as much as 20% of the variation in the circulating levels of apoB and triglycerides between subjects. The driving force may be an influence of adipose triglyceride turnover on apoB levels, although it remains possible that local inflammation in adipose tissue could also be involved**.**

## Acknowledgment

The skillful technical assistance of Kerstin Wåhlen is greatly appreciated. P.A. and K.F. analyzed data and K.S. wrote the first version of the article. K.S. and S.B. were responsible for the turnover data and wrote, together with K.F. and P.A., the subsequent versions of the article.
